# Genome-Wide Identification and Evolution Analysis of R2R3-MYB Gene Family Reveals S6 Subfamily R2R3-MYB Transcription Factors Involved in Anthocyanin Biosynthesis in Carrot

**DOI:** 10.3390/ijms231911859

**Published:** 2022-10-06

**Authors:** Ao-Qi Duan, Shan-Shan Tan, Yuan-Jie Deng, Zhi-Sheng Xu, Ai-Sheng Xiong

**Affiliations:** State Key Laboratory of Crop Genetics and Germplasm Enhancement, Ministry of Agriculture and Rural Affairs Key Laboratory of Biology and Germplasm Enhancement of Horticultural Crops in East China, College of Horticulture, Nanjing Agricultural University, 1 Weigang, Nanjing 210095, China

**Keywords:** carrot, R2R3-MYB family, transcription factor, anthocyanin, evolution, expression profile

## Abstract

The taproot of purple carrot accumulated rich anthocyanin, but non-purple carrot did not. MYB transcription factors (TFs) condition anthocyanin biosynthesis in many plants. Currently, genome-wide identification and evolution analysis of R2R3-MYB gene family and their roles involved in conditioning anthocyanin biosynthesis in carrot is still limited. In this study, a total of 146 carrot R2R3-MYB TFs were identified based on the carrot transcriptome and genome database and were classified into 19 subfamilies on the basis of R2R3-MYB domain. These R2R3-MYB genes were unevenly distributed among nine chromosomes, and *Ka*/*Ks* analysis suggested that they evolved under a purified selection. The anthocyanin-related S6 subfamily, which contains 7 MYB TFs, was isolated from R2R3-MYB TFs. The anthocyanin content of rhizodermis, cortex, and secondary phloem in ‘Black nebula’ cultivar reached the highest among the 3 solid purple carrot cultivars at 110 days after sowing, which was approximately 4.20- and 3.72-fold higher than that in the ‘Deep purple’ and ‘Ziwei’ cultivars, respectively. The expression level of 7 MYB genes in purple carrot was higher than that in non-purple carrot. Among them, *DcMYB113* (*DCAR_008994*) was specifically expressed in rhizodermis, cortex, and secondary phloem tissues of ‘Purple haze’ cultivar, with the highest expression level of 10,223.77 compared with the control ‘DPP’ cultivar at 70 days after sowing. *DcMYB7* (*DCAR_010745*) was detected in purple root tissue of ‘DPP’ cultivar and its expression level in rhizodermis, cortex, and secondary phloem was 3.23-fold higher than that of secondary xylem at 110 days after sowing. Our results should be useful for determining the precise role of S6 subfamily R2R3-MYB TFs participating in anthocyanin biosynthesis in carrot.

## 1. Introduction

Carrot (*Daucus carota* L.), an annual or biennial herb vegetable crops, is one of the 10 vegetable crops grown worldwide, and has a long history of cultivation. According to its taproot color, they can be divided into five types: white, yellow, orange, red and purple carrot [1,2,3,4]. Non-purple carrot is produced by a mutation of purple carrot. Non-purple carrots are well known for their orange pigmentation and carotenoid accumulation [5]. Purple carrot can be divided into solid purple carrot type and tissue-specific purple carrot types [6,7,8,9]. Nowadays, purple carrots are popular among consumers because they are an excellent source of anthocyanins. As the anthocyanin group, they accumulate in fleshy roots, as well as in flowers, petioles and fruits. Carrots can be used to produce anthocyanin-rich concentrate for the pigment industry [10,11].

MYB transcription factors, as one of the largest transcription factor families in plants, are characterized at most four repetitive sequences (R), each of which is about 53 amino acids, forming H1, H2 and H3 α-helices [12]. In each repeat, H2 and H3 helices form helix-turn-helix structure respond for the DNA interaction of MYB domain [13]. According to the repeat number, MYB TFs were divided into four types: 1R-MYB, R2R3-MYB, 3R-MYB and 4R-MYB [14]. Among the four types, R2R3-MYB transcription factor is the most common one, including two tandem repeats [15]. R2R3-MYB usually forms a complex with bHLH and WD40 proteins, which directly acts on structural genes of anthocyanin synthesis pathway in plants, thus affecting anthocyanin synthesis and accumulation [16,17,18]. MYB and MYB-related sequences are available in the Plant Transcription Factor Database [19]. The classification of R2R3-MYB TF members and the anthocyanins accumulation have been reported in many species. The whole genome sequences of R2R3-MYB TFs in different species have also been identified, such as *Arabidopsis thaliana* (126 R2R3-MYB proteins) [20], *Gossypium raimondii* (205 R2R3-MYB proteins) [21], soybean (244 R2R3-MYB proteins) [22], *Oryza sativa* (88 R2R3-MYB proteins) [23], apple (222 apple typical R2R3 MYB proteins) [24], Chinese Pear (105 R2R3-MYB proteins) [25] and tea plants (122 CsR2R3-MYB proteins) [26].

Anthocyanins are water-soluble natural pigments widely distributed in fruits and vegetables, with pH-dependent color changing from red to purple and then to blue. They are important secondary metabolites of plants, are synthesized in the cytosol and stored in vacuoles. Anthocyanins mainly contain six common aglycones and various types of glycosylations and acylations [27]. Over 600 kinds of anthocyanins have been identified, and the edible anthocyanins in nature include colored fruits and vegetables, such as apples, cherries, grapes, carrots, eggplant, celery, water dropwort and purple cabbage [28,29,30,31,32,33,34]. Anthocyanins are usually used as food colorants, which are beneficial to human health and have anti-cancer and anti-oxidation properties [29,35]. As a natural colorant, it is enjoying increasing loved by producers and consumers [30]. Among plants, pigments play an important role in attracting insects for seed transmission and pollination and improving the ability to resist abiotic and biological stresses.

In this study, to obtain further understanding in differences of anthocyanin synthesis between purple and non-purple carrot, we obtained the R2R3-MYB TFs members based on the carrot transcriptome and genome database, which are related to anthocyanin synthesis in carrot. Then, phylogenetic relationships, physicochemical properties, and anthocyanin contents of different color carrots were detected and analyzed. Expression patterns of seven selected *DcMYB* genes of the S6 subfamily in the taproots of six purple and three non-purple carrot cultivars were determined. This study will help establish the regulatory molecular mechanism of R2R3-MYB gene and pave the way for future functional research on anthocyanin in carrot.

## 2. Results

### 2.1. Evolution of MYB TFs of Carrot in Different Species

**Figure 1:**
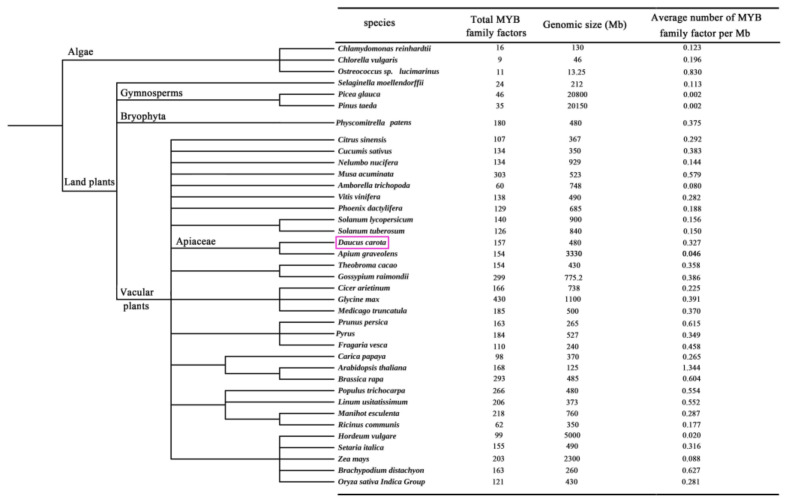
Summary of MYB TFs among plants. *Daucus carota* was highlighted with purple box.

As shown in the Figure 1, the number of MYB TFs varies from several to several hundred in different species. The number of MYB family transcription factors in higher plants is generally higher than that in lower algae, which may be due to the replication and expansion of MYB TFs during the evolution of plants. The number of MYB family transcription factors in *Musa acuminata*, *Gossypium raimondii*, *Glycine max*, *Brassica rapa* and *Populus trichocarpa* was greater than 200. Compared with celery (3.33 Gb) [34,36], carrot has a smaller genome (~0.5 Gb) [37], but a similar number of MYB transcription factors.

### 2.2. Classification and Phylogenetic Analysis of R2R3-MYB TFs in D. carota and A. thaliana and Analyses of Chromosomal Locations

**Figure 2:**
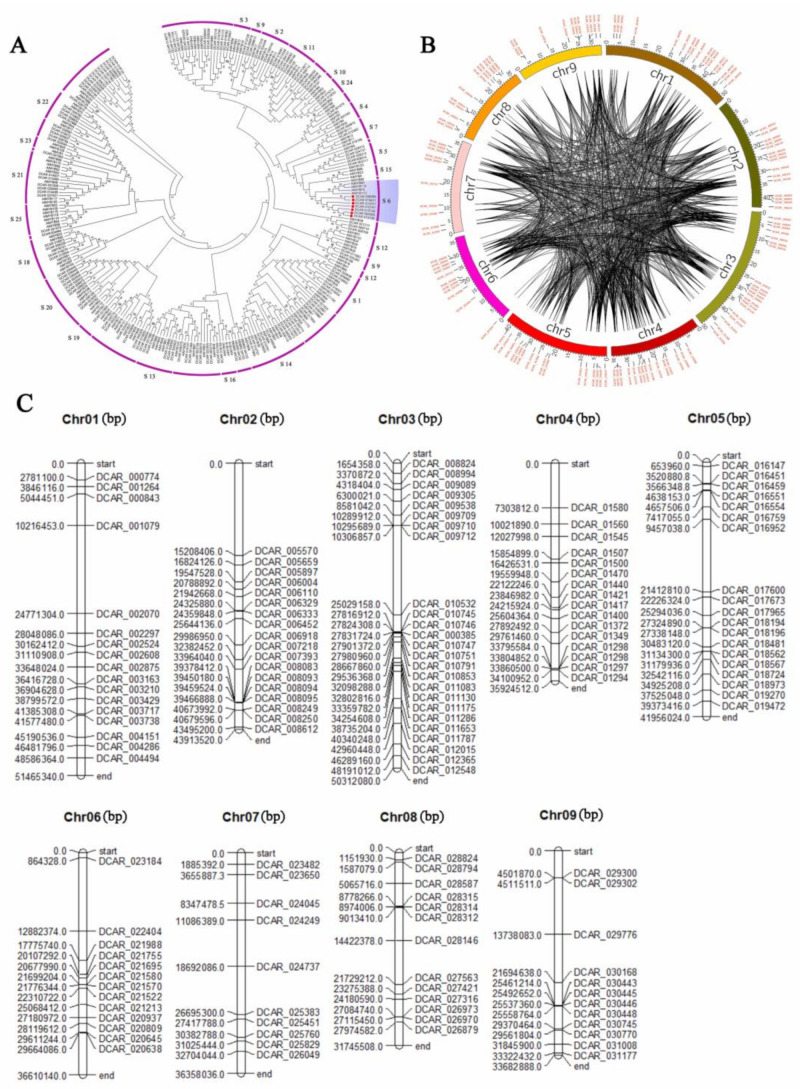
Phylogenetic tree of R2R3-MYB TFs of *Arabidopsis* and carrot and chromosomal determination and collinearity analysis of R2R3-MYB genes in carrot. Phylogenetic trees were constructed from the R2R3-MYB protein sequence of carrot and *Arabidopsis* (**A**). The phylogenetic trees were established by the neighbor-joining (NJ) method. (**B**,**C**) represent the carrot chromosomes. The black lines represent collinear gene pairs. The genes were mapped onto different chromosomes and the numbers along the chromosome boxes represent sequence lengths in megabases.

**Figure 3:**
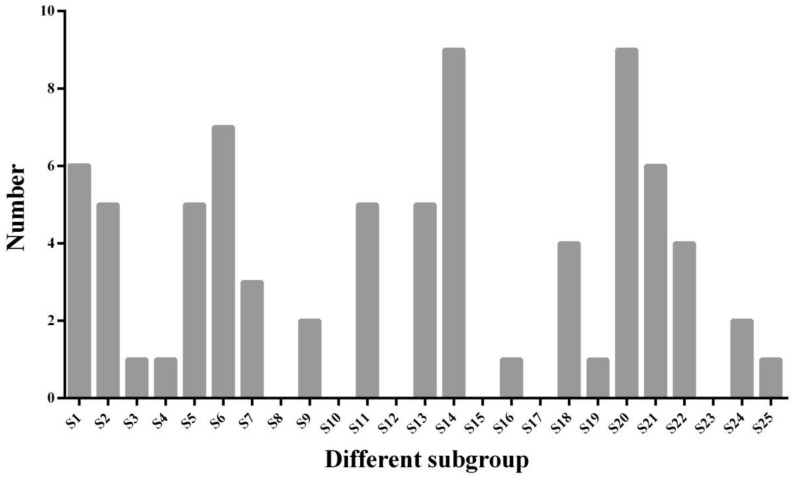
Distribution of carrot R2R3-MYB TFs subfamilies. R2R3-MYB TFs subfamilies represented on the Abscissa. Ordinate represents the number of each subfamily member.

Results showed that 146 R2R3-MYB TFs were identified based on carrot genome. The amino acid sequences of R2R3-MYB TFs in carrot and *A. thaliana* were aligned to draw a phylogenetic tree (Figure 2 and Supplementary Table S1). According to the evolutionary relationship of R2R3-MYB TFs between carrot and *A. thaliana*, MYB TFs of carrot were divided into 19 subfamilies (Figure 3). The number of MYB TFs in S14 and S20 subfamilies was the largest, with 9 in each subfamily. The second is the S6 subfamily, which has 7 TFs. In *A. thaliana*, the functions of R2R3-MYB in different subfamilies are widely different than in plants. *A. thaliana* S6 subfamily members include AtMYB75, AtMYB90, AtMYB113 and AtMYB114, which plays a regulatory role in anthocyanin biosynthesis [13,38,39]. Similarly, R2R3-MYB TFs in the S6 subfamily of carrot related to anthocyanin biosynthesis, including DcMYB6 (DCAR_000385), DcMYB7 (DCAR_010745), DcMYB8 (DCAR_010746), DcMYB9 (DCAR_010747), DcMYB11 (DCAR_010751), DcMYB113 (DCAR_008994) and DcMYB016 (DCAR_016451). According to the starting position of the R2R3-MYB gene in chromosomes, it was found that *DcMYB* genes were unevenly distributed among 9 carrot chromosomes (Figure 2B). Some *DcMYB* genes cannot eventually be mapped on any chromosome. Chromosome 3 contained the most *DcMYB* genes (20), followed by chromosome 1 (17), and the lowest numbers were found on chromosome 7 (10). More genes were observed at the bottom of chromosomes 1, 2, 3 and 6, and the genes on chromosomes 7 and 8 were evenly distributed.

### 2.3. Conserved Motifs of R2R3-MYB Proteins Based on the Analysis of MEME

**Figure 4:**
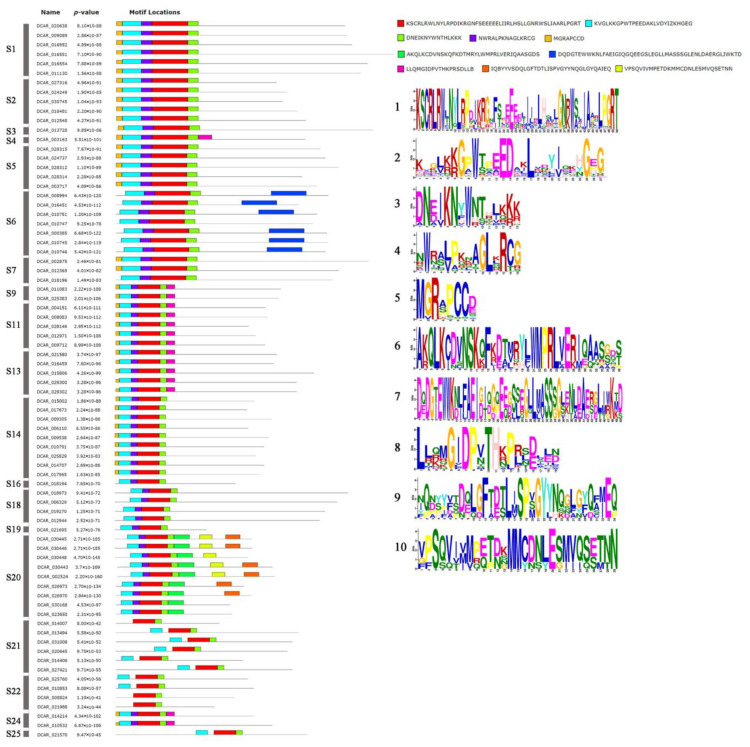
Sequence logos of conserved domains of R2R3-MYB proteins in carrot.

According to the evolutionary relationship of R2R3-MYB TFs in carrot and *A. thaliana*, R2R3-MYB TFs in carrot were divided into different subfamilies. The conserved domains of all class members were analyzed by online software MEME. Through online analysis, a total of 10 conserved motifs were obtained, and the distribution of conserved motifs in different subfamilies is shown in the Figure 4. Closely related genes had the same motif compositions, suggesting that there were functional similarities between MYB proteins. Motifs 7 only exists in the S6 subfamily. Motif 9 only exists in the S20 subfamily, and the S20 subfamily contained the most motifs. Reports on the regulatory mechanism of *DcMYBs* in different subfamilies of carrot are still limited. Based on the homology with *Arabidopsis thaliana*, we infer the possible functions of *DcMYBs* subfamilies. Subfamilies S1 and S4 may participate in the response to biotic and abiotic stresses [40,41]. Subfamily S2 may control the biosynthesis of proanthocyanidins (PAs) [42,43]. Subfamily S3 and subfamily S21 may be involved in cell wall biosynthesis [13,44]. Subfamily S6 controls anthocyanin biosynthesis in plant tissues [13]. Subfamily S7 may control flavonol biosynthesis [45]. Subfamily S13 may play a role in influencing lignin deposition and stomatal aperture [46]. Subfamily S22 was proposed to regulate lateral root formation [13] and subfamily S25 was proposed to play roles in embryogenesis [47].

### 2.4. Selection Pressure in Carrot

**Figure 5:**
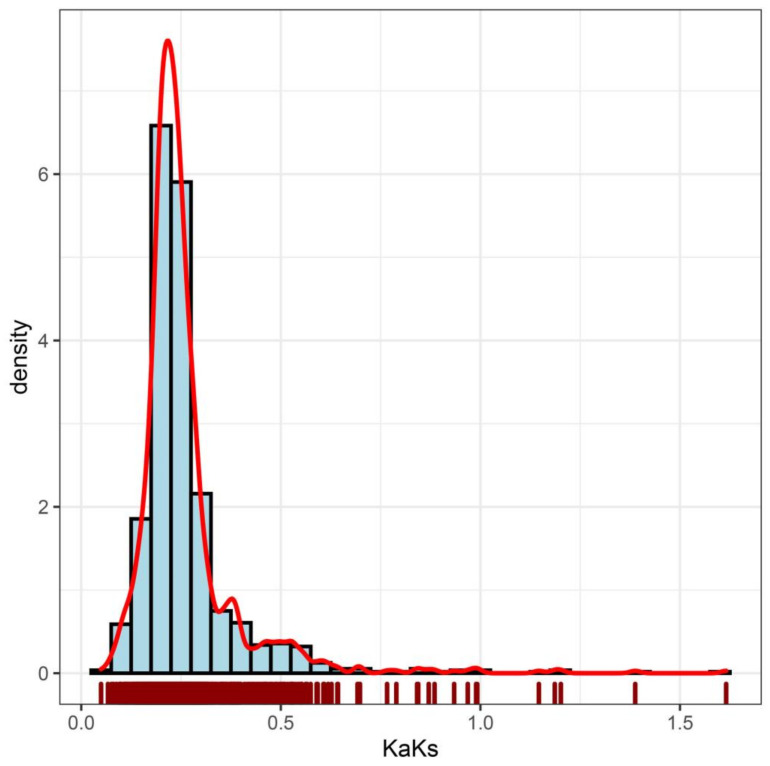
*Ka*/*Ks* density distribution in carrot.

The collinearity relationships of the *MYB* genes in carrot chromosomes are presented in Supplementary Tables S2 and S3. Positive selection promotes the evolution of animals and plants by accumulating favorable mutations, while purified selection promotes the evolution of animals and plants by eliminating harmful mutations. To determine which selection pressure promoted the evolution of R2R3-MYB gene in carrot, we used the CDS sequence of R2R3-MYB gene to calculate *Ka*, *Ks* value and *Ka*/*Ks* ratio (Figure 5 and Supplementary Table S2). The *Ks* value for a clear majority of R2R3-MYB paralog gene pairs in carrot is higher than *Ka*, and the *Ka*/*Ks* value of paralog gene pairs is mostly less than 1, indicating that the evolution of the R2R3-MYB gene in-species is mainly performed via purified selection. The *Ka*/*Ks* ratio is mostly concentrated in the range of 0.1–0.4, indicating strong purified selection.

### 2.5. Anthocyanin Content in Different Root Tissues of Nine Carrot Cultivars at Different Development Stages

**Figure 6:**
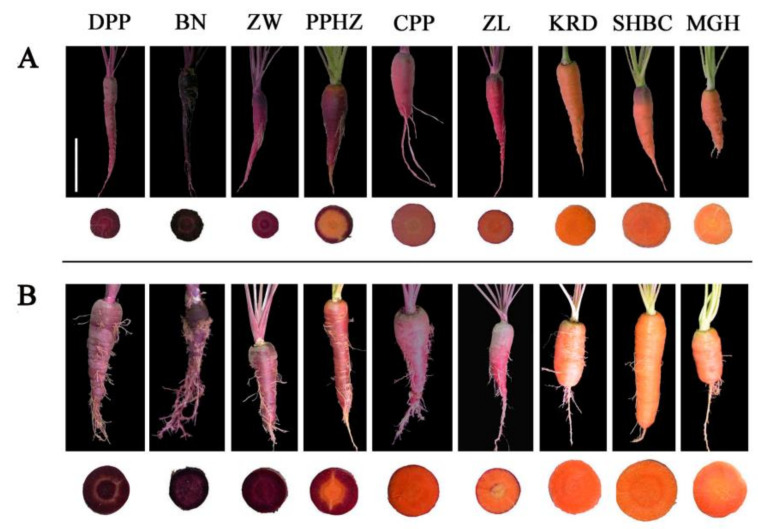
The color of cross-sections and the taproots of nine purple and non-purple carrots cultivars at two different stages. (**A**) represents the purple and nonpurple carrots grown for 70 days. (**B**) represents the purple and nonpurple carrots grown for 110 days. Cultivar abbreviations: DPP, Deep purple; BN, Black nebula; ZW, Ziwei; PPHZ, Purple haze; CPP, Cosmic purple; ZL, Zilong; KRD, Kurodagosun; SHBC, Sanhongbacun; MGH, Meiguihong. White bar represents 5 cm.

**Figure 7:**
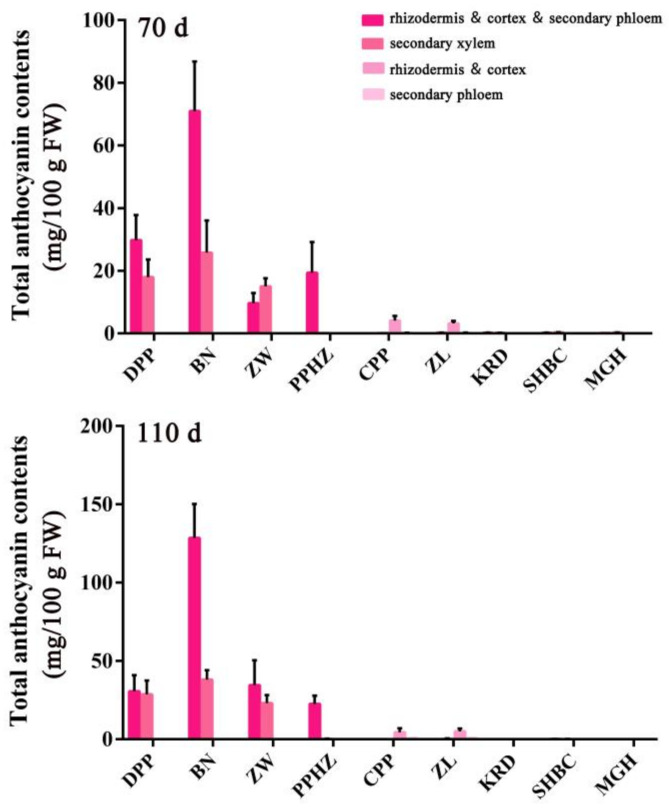
Total anthocyanin contents in the taproot of nine carrot cultivars at two. different stages. Error bars represent standard deviation (SD) of three replicates and are computed as cyanidin 3-O-galactoside equivalents.

Anthocyanins were distributed in different parts of carrot taproots, and it made the taproot appear purple and dark purple color (Figure 6). The anthocyanins of PPHZ taproots only accumulated in the rhizodermis, cortex, and secondary phloem, and the purple area increased with the development stage. Anthocyanins accumulated in the rhizodermis and cortex of CPP and ZL but did not exist in secondary phloem and secondary xylem (Figure 6). No purple or dark purple coloring was detected in KRD, MGH, SHBC taproots. Anthocyanins accumulated in rhizodermis, cortex, and secondary phloem and secondary xylem of DPP, BN and ZW carrot taproots in 70 and 110 days after sowing. Total anthocyanin content in different parts of six purple carrot cultivars taproots (DPP, BN, ZW, PPHZ, CPP, ZL) increased significantly with development process, and the anthocyanin-increased efficiency of taproot in solid purple carrots was higher than others (Figure 7). Among these six purple carrot cultivars, the anthocyanin accumulation of BN in the taproot was the highest at the two periods. At the 70 and 110 days after sowing, total anthocyanin accumulation in rhizodermis, cortex, and secondary phloem reached 71.00 and 128.47 mg/100 g fresh weight (fw) respectively, while that in the secondary xylem reached 25.78 and 38.04 mg/100 g fw respectively (Figure 7). No anthocyanin accumulation was detected in MGH, KRD and SHBC taproots. There was no significant difference in the anthocyanin accumulation between the rhizodermis and cortex of CPP and ZL, and the increase efficiency in the two development stages was lower. At the 70 days after sowing, the anthocyanin contents in the rhizodermis and cortex of CPP and ZL were 4.11 and 3.23 mg/100 g fw respectively, while 110-day-old rhizodermis and cortex’ anthocyanin values were 4.50 and 5.04 mg/100 g fw, respectively.

### 2.6. Expression Profiles of DcMYB TFs in S6 Subfamily of Different Carrot Cultivars and Position of DcMYB Genes in S6 Subfamily on Chromosome

**Figure 8:**
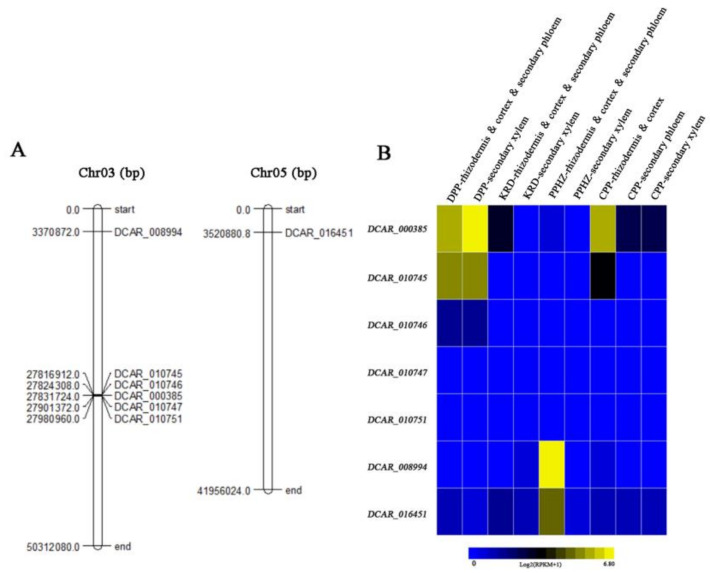
Site of MYB TFs in S6 subfamily on chromosome and transcript abundances of *DcMYB* genes in four different tissue parts of *D. carota* cvs. DPP, PPHZ, CPP and KRD. (**A**) represents the site of MYB TFs. Heat map was constructed based on the log2 (RPKM + 1) transformation values (**B**), and bar notes represent different expression levels. Yellow represents high expression level, and blue represents little expression level.

We selected 7 *DcMYB* genes in S6 subfamily and mapped their location onto chromosomes (Figure 8A). *DcMYB016* (*DCAR_016451*) was located on chromosome 5 and the rest on chromosome 3. *DcMYB113* (*DCAR_008994*) on chromosome 3 was far away from the other five transcription factors. Based on the carrot transcriptome data with different colors, transcripts of R2R3-MYB TFs in S6 subfamily were analyzed. The heat map relying on log2 (RPKM + 1) transformation value was constructed (Figure 8B). Yellow represents high expression level and blue represents low expression level. The results of transcriptome analysis showed that these *DcMYB* genes of S6 subfamily expression levels were different in carrot cultivars. *DcMYB7* (*DCAR_010745*) was highly expressed in purple tissues, but hardly expressed in non-purple carrot. *DcMYB6* (*DCAR_000385*) had the higher taproot mRNA levels in purple carrot cultivars than non-purple carrot. *DcMYB113* (*DCAR_008994*) was highly expressed in rhizodermis, cortex, and secondary phloem of PPHZ, but less expressed in other cultivars.

### 2.7. Relative Transcript Levels of S6 Subfamily DcMYBs in Different Root Tissues of Nine Carrot Cultivars

**Figure 9:**
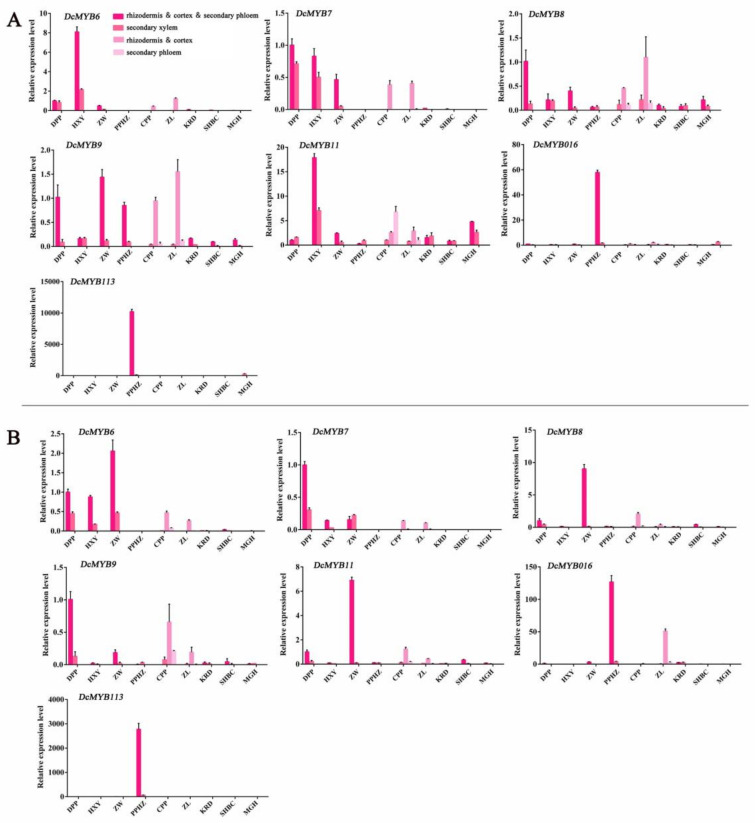
Expression profiles of S6 subfamily DcMYB genes (DcMYB6 (DCAR_000385), DcMYB7 (DCAR_010745), DcMYB8 (DCAR_010746), DcMYB9 (DCAR_010747), DcMYB11 (DCAR_010751), DcMYB016 (DCAR_016451) and DcMYB113 (DCAR_008994)) in different root tissues of carrot at two growth stages. (**A**) represents expression profiles of *DcMYB* genes in purple and nonpurple carrots at 70 days after sowing. (**B**) represents expression profiles of *DcMYB* genes in purple and nonpurple carrots at 110 days after sowing. The relative gene expression was calculated with the 2^−ΔΔCT^ method. Error bars represent standard deviation (SD) of three replicates.

To better analyze the response of S6 subfamily members in root tissue of carrot cultivars, we sampled different parts in taproots of 9 purple and non-purple carrot cultivars (Figure 9). For RT-qPCR assay, 7 *DcMYB* genes (*DcMYB6* (*DCAR_000385*), *DcMYB7* (*DCAR_010745*), *DcMYB8* (*DCAR_010746*), *DcMYB9* (*DCAR_010747*), *DcMYB11* (*DCAR_010751*), *DcMYB113* (*DCAR_008994*) and *DcMYB016* (*DCAR_016451*)) from S6 subfamily were selected to determine the expression profiles of different root tissue during two development stages. At 70 days after sowing, the expression levels of *DcMYB6* (*DCAR_000385*) and *DcMYB7* (*DCAR_010745*) were higher in all purple carrot root tissue, and the transcripts of secondary xylem were lower than those of rhizodermis, cortex, and secondary phloem tissues. In ZL and CPP, the two gene transcripts, *DcMYB6 (DCAR_000385)* and *DcMYB7 (DCAR_010745)*, were detected in the rhizodermis and cortex. *DcMYB113 (DCAR_008994)* and *DcMYB016 (DCAR_016451)* had the highest expression in the rhizodermis, cortex, and secondary phloem of PPHZ, which was consistent with the gene expression at 110 days after sowing.

## 3. Discussion

Carrot is an important vegetable crop of *Apiaceae* family. In addition, celery and water dropwort and coriander also belong to common *Apiaceae* plants [4,48,49]. In addition to having rich anthocyanins [7,11,50], carrot has many active compounds, including carotenoids, volatile oil, vitamins, dietary fiber [4,48] and other nutrients.

Anthocyanins are very important secondary metabolites in many plants with functions on providing antioxidant protection and scavenging free radical. Anthocyanins are one of the most health-protective ingredients in the human diet. Eating fruits and vegetables rich in anthocyanins is conducive to improving human health, and also can effectively prevent cardiovascular diseases and improve blood sugar balance [30]. The high expression of *MYB* gene in fruits and vegetables plays an important role in anthocyanin biosynthesis of plants. There are few studies on the genome-wide classification of R2R3-MYB gene family and its transcription in different root tissues of carrot. RT-qPCR was used to analyze the gene expression of S6 subfamily in purple and non-purple carrot at different developmental stages, which is helpful to further understand the molecular regulation mechanism of DcMYB transcription factor on carrot root development. These R2R3-MYB gene transcript levels were normalized to *DcActin1* [51].

*MYB* gene is widely distributed in plants and constitutes one of the largest transcription factor families. It acts a significant role in color accumulation, growth metabolism and stress response of plant organs. Since the first MYB transcription factor C1 was found in maize, with the development of genome sequencing and bioinformatics, more and more *MYB*-functional genes have been studied in different species [20,52,53,54,55]. In this study, we identified 146 R2R3-MYB factors, analyzed their phylogeny with 126 R2R3-MYB genes from *A. thaliana*, and divided them into 19 subfamilies. According to homology alignment, DcMYB TFs with similar motifs were divided into the same branch. The sequence structure of R2R3-MYB protein in different subfamilies is usually connected to biological function, and R2R3-MYB protein in S4-S7 subfamilies is related to phenylalanine metabolism regulating plant metabolite synthesis [38,39,42,56]. Consistent with sequence alignment and phylogenetic tree analysis, MEME analysis showed that the conserved domains of R2R3-MYB transcription factors in the same subfamilies were similar, indicating that these conserved motifs were associated with the regulatory functions of different subfamilies [39]. The number of R2R3-MYB transcription factors in higher plants is higher than that in lower plants, which may be due to the replication of plant genome during evolution. Besides, some *DcMYBs* could not be mapped to any chromosome, which might be due to the quality of the carrot genome sequence or a high level of heterozygosity [25]. High levels of heterozygosity within an individual might be an indication of low sample quality. The *Ka*/*Ks* ratios of clear R2R3-MYB genes in carrot indicated that the R2R3-MYB gene family in *Apiaceae* has generally undergone purifying selection and highly conserved evolution. Parts of the CDS of *R2R3-MYBs* have undergone positive selection, indicating that new gene functions might have been acquired. Such genes are rapidly evolving genes recently, which may be of great significance to the evolution of species.

Tandem and segmental duplication events had been hypothesized as the leading driving mechanism for the expansion and chromosomal organization into clusters of MYB gene families [57]. R2R3-MYB family members had been commonly found in gene clusters in genomes of many plant species. The S6 cluster is related to anthocyanin accumulation in *A. thaliana* and is the main subfamily of anthocyanin accumulation in *A. thaliana*, which primarily includes AtMYB75, AtMYB90, AtMYB113 and AtMYB114 [16,58]. Similar to *Arabidopsis*, MYB members classified as S6-MYB type in Petunia and tomato regulated anthocyanin biosynthesis [38,39]. Phylogenetic tree analysis of R2R3-MYB transcription factors in carrot and *A. thaliana* showed that 7 MYB TFs and *A. thaliana* S6 subfamily clustered together. DcMYB proteins in the same branch are highly correlated and may have similar functions. In all subfamilies, there were 9 MYBs in the subfamilies S14 and S20 that contained the most MYBs. The S14 subfamily is related to the formation of meristem in *A. thaliana*, among them, three R2R3-MYBs (*RAX* genes) were designated as regulators of axillary meristem. The *Rax* gene controls the early stage in the initiation of axillary meristem [59]. S20 subfamily *AtMYB62* was involved in response to phosphate starvation [60]. There were no R2R3-MYB members found in the S8, S10, S12, S15, S17 and S23 R2R3-MYB subfamilies. These results may imply the special characteristics R2R3-MYB genes in carrot species.

The expression pattern of R2R3-MYB genes in different developmental stages of carrot was studied by transcriptome analysis [61]. A total of 7 R2R3-MYB genes related to anthocyanin accumulation were tested. Significant changes in the expression levels of all of the members in S6 subfamily were detected. Similarly, these data agree with the previous analysis described above, suggesting that several genes in S6 subfamily had been confirmed to be participants in the anthocyanin biosynthesis of carrot. Research showed that *DcMYB6* (*DCAR_000385*) had high transcript levels in the anthocyanin-pigmented carrot tissues with purple color, and it was detectable in the non-purple carrot cultivars. *DcMYB6* (*DCAR_000385*) can induce anthocyanin biosynthesis of *A. thaliana* [9]. *DcMYB7* (*DCAR_010745*), designated as the *DcMYB113-like* gene in the carrot genome, was always associated with purple root pigmentation of all purple carrots [6,7,11,17,58,62,63]. This corresponds to our transcriptional and heatmap values. In addition, *DcMYB016* (*DCAR_016451*) and *DcMYB113* (*DCAR_008994*) in the rhizodermis, cortex, and secondary phloem of PPHZ were significantly higher than that in other root tissues, suggesting that they may be a key gene regulating anthocyanin synthesis in rhizodermis, cortex, and secondary phloem. Previous studies on anthocyanin accumulation of carrot R2R3-MYB TFs have been reported, but there is no systematic classification of R2R3-MYB TF family. Systematic classification and transcriptional studies will provide us valuable tools to explore its potential application in carrot molecular breeding.

## 4. Materials and Methods

### 4.1. Plant Materials and Growth Conditions

Three solid purple root types (‘Deep purple’ (DPP), ‘Black nebula’ (BN) and ‘Ziwei’ (ZW)), three partial purple root types (‘Purple haze’ (PPHZ), ‘Cosmic purple’ (CPP) and ‘Zilong’ (ZL)) and three non-purple carrot cultivars (‘Kurodagosun’ (KRD), ‘Sanhongbacun’ (SHBC) and ‘Meiguihong’ (MGH)) were used as experimental materials and planted in the artificial climate room of Nanjing Agricultural University (32°04′ N, 118°85′ E, Nanjing, China). Organic soil, vermiculite and perlite (2:2:1, *v*/*v*) were used as substrates. The artificial climate chamber was set at 25 °C for 16 h in the daytime and 16 °C for 8 h in the evening, and the light intensity was 320 μmol m^−2^ s^−1^, and the relative humidity was set at 75%. After 70 and 110 days after sowing plant growth, different tissues (rhizodermis, cortex, secondary phloem and secondary xylem) of carrot taproot were sampled. One part was used to extract RNA, and the other part was used to determine anthocyanin content. Three independent biological replicates of each carrot plant sample were prepared.

### 4.2. Identification of R2R3-MYB TFs in Carrot

The sequences of R2R3-MYB TFs in carrot were extracted based on the carrot genome database [64] and plant genome database (https://phytozome.jgi.doe.gov/pz/portal.html) (accessed on 1 May 2021) [52]. A total of 126 R2R3-MYB TFs sequences from A. thaliana were downloaded from the website TAIR (http://www.arabidopsis.org/) (accessed on 1 May 2021) [65]. The MYB TFs of other species are derived from Plant Transcription Factor Database [19]. The obtained candidate sequences are identified through Hmmer and Pfam number (PF00249), and the parameters were the default value.

### 4.3. Constructions of Phylogenetic Tree, Evolutionary Analysis and Sequence Features Analysis

Homologous genes were further analyzed by BLASTp search. Multiple sequences of R2R3-MYB protein from *A. thaliana* and carrot were aligned by Cluster W [66]. Then a phylogenetic tree was constructed by neighbor-joining method (bootstrap value of 1000) with MEGA 7 [67]. Position of MYB TFs in S6 subfamily on chromosome was anchored to a physical map with Mapchart v2.32. The conserved domains of R2R3-MYB TFs were analyzed by MEME search. Evolutionary relationship was obtained via online software (http://www.ncbi.nlm.nih.gov/Taxonomy/CommonTree/wwwcmt.cgi) (accessed on 1 May 2021). The species evolution diagram was drawn by online software (http://itol.embl.de/upload.cgi) (accessed on 1 May 2021). The heat map of *DcMYBs* candidate gene expression in carrot was established by HemI 1.0 software (http://hemi.biocuckoo.org/faq.php) (accessed on 1 May 2021) [68].

### 4.4. Collinearity and Non-Synonymous Substitution Rate (Ka)/Synonymous Substitution Rate (Ks) Analyses

To further understand the structural information of R2R3-MYB gene family in carrot family, we analyzed the chromosomal localization and collinearity of R2R3-MYB gene family in carrot. MCScanX software was used to analyze collinearity of carrot R2R3-MYB gene family. In addition, the homologous gene pairs of R2R3-MYB gene were extracted and visualized with TBtools software.

In order to identify the selection pressure on R2R3-MYB genes in the process of evolution, we calculated the *Ka* and *Ks*. The ratio of *Ka* and *Ks* can be used to measure the selection pressure. Firstly, the coding sequence and nucleotide sequence of R2R3-MYB gene of carrot were compared with ParaAT2.0 software [69], and then *Ka*, *Ks* and *Ka*/*Ks* values were calculated by *KaKs*_calculator2.0 software [70,71]. When *Ka*/*Ks* > 1, it indicates positive selection. When *Ka*/*Ks* < 1, it indicates purified selection. When *Ka*/*Ks* = 1, it indicates neutral evolution.

### 4.5. Anthocyanin Measurement

Total anthocyanins were extracted from different tissues of carrot root by methanol–HCl method [3,9]. The fresh weight of different tissues of carrot root was determined after sampling, and then ground to powder in liquid nitrogen. The milled sample was transferred to the extract (50 % methanol, 49.9 % double distilled water, 0.1 % HCl, V/V) for the whole night. The 200 μL supernatant was absorbed to determine the absorbance for quantitative analysis at 530 nm, 620 nm and 650 nm. Optical density of anthocyanin OD_λ_ = (OD_530_ − OD_620_) − 0.1 × (OD_650_ − OD_620_). The total anthocyanin content was expressed by the weight of cyanidin 3-O-galactoside per 100 g plant fresh weight [33]. Three biological replicates and three technical replicates were set for the extraction and determination of anthocyanin.

### 4.6. RT-qPCR Analysis

A total of 7 *DcMYB* genes of S6 subfamily were used for RT-qPCR analysis (Table 1). The specific primers were designed by Primer Premier 6.0 and synthesized by Genscript Inc. (Nanjing, China). Real-time quantitative PCR was performed using the instructions of the SYBR Premix *Ex Taq* kit (TaKaRa, Dalian, China). Using a 20 μL system, each PCR reaction system contained 2.0 μL of cDNA, 0.4 μL of forward and reverse quantification primers, 10 μL of SYBR Premix *Ex Taq*, and 7.2 μL of ddH_2_O. The relative gene transcript levels were normalized to *DcActin1* [51] and calculated by the formula 2^−ΔΔCT^ [72]. Each PCR reaction were performed with three independent biological replicates.

### 4.7. Statistical Analysis

The column charts were drawn with Graphpad Prism 6.0 software. Bars represent the mean values of three biological replicates ± standard deviation.

## Figures and Tables

**Table 1:** The primer sequences used for RT-qPCR of S6 subfamily *DcMYB* genes.

Gene ID	Forward Primer (5’→3’)	Reverse Primer (5’→3’)
*DCAR_000385*	AGTGGCATCCTCAAGTGGTTCA	CCCAAATGTCACTCCAGCAACT
*DCAR_010745*	AGCGGCAACGACATTAACAACA	TTCATCTGGTAAGGCGGTGGTT
*DCAR_010746*	GCAGCAGCAACATCAACAACGA	TTCATCTGGTAAGGCGGTGGTT
*DCAR_010747*	TGCCACTACTTGTACCGCTACC	TCCTCCACCACTCGTTTCCATC
*DCAR_010751*	AAGCCTGTTCCGCAGACCTTAA	GGACGCCACTTGAGGACACAT
*DCAR_008994*	AGTGGCACCTTGTTCCTCAGAG	GCTGGCAATGATGGCTTCTTGT
*DCAR_016451*	GTGGCACCTTGTTCCTCAGAGA	TCGTCATTCAGTGGCAGTGTTG
*DcActin1*	CGGTATTGTGTTGGACTCTGGTGAT	CAGCAAGGTCAAGACGGAGTATGG

## Data Availability

All the other data sets supporting the conclusions of this article are included within the article and its additional files.

## Supplementary Materials

The following supporting information can be downloaded at: https://www.mdpi.com/article/10.3390/ijms231911859/s1. Table S1. The sequence information of R2R3-MYB TFs in carrot and *A. thaliana* identified in this study. Table S2. Information of Ka/Ks of the MYB genes in carrot chromosomes. Table S3. Information of collinear MYB gene pair in carrot chromosomes.
